# Effect of Atmospheric Cold Plasma Treatment on the Microorganism Growth, Diversity, and Quality of Coconut Water During Refrigerator Storage

**DOI:** 10.3390/foods14152709

**Published:** 2025-08-01

**Authors:** Lixian Zeng, Wenyue Gu, Yuanyuan Wang, Wentao Deng, Jiamei Wang, Liming Zhang

**Affiliations:** School of Food Science and Engineering, Hainan University, Haikou 570228, China; zeng18218841215@163.com (L.Z.); guweny2022@126.com (W.G.); yuanyuanwang0224@163.com (Y.W.); w18316478401@163.com (W.D.)

**Keywords:** cold plasma, coconut water, physicochemical properties, bacteria diversity, microorganism amount

## Abstract

To study the effect of cold plasma (CP) on the refrigerator shelf life of coconut water, microorganism growth and diversity and physicochemical properties were investigated. Results indicated that CP treatment did not cause significant color changes in coconut water, with turbidity remaining lower than the control even after 6 days of storage. Enzymatic activity analysis revealed reduced polyphenol oxidase (PPO) and peroxidase (POD) levels in treated samples. Specifically, the 12 s CP treatment resulted in the lowest antioxidant capacity values: 15.77 Fe^2+^/g for ferric reducing antioxidant power (FRAP), 37.15% for DPPH radical scavenging, and 39.51% for ABTS^+^ radical scavenging. Microbial enumeration showed that extended CP treatment effectively inhibited the growth of total viable counts, psychrophilic bacteria, lactic acid bacteria, and yeast. High-throughput sequencing identified *Leuconostoc*, *Carnobacterium*, and *Lactobacillus* as the dominant bacterial genera. During storage, *Carnobacterium* was the primary genus in the early stage, while *Leuconostoc* emerged as the dominant genus by the end of the storage period. In summary, CP as an effective non-thermal technology was able to maintain quality and antioxidant capacity, inhibit microbial growth, and delay the spoilage in coconut water to help extend the refrigerated shelf life of the product.

## 1. Introduction

Coconut water is low in calories and fat, yet rich in minerals, vitamins, and amino acids, contributing to its popularity among consumers [1,2,3]. The coconut water on the market today mainly undergoes treatment with ultra-high temperatures for instantaneous sterilization. However, coconut water is highly sensitive to heat and light, which can lead to discoloration, off-flavors, and microbial contamination, resulting in spoilage and loss of commercial value [4]. Therefore, to maintain the favorable sensory quality and physicochemical properties of coconut water and extend its shelf life, non-thermal sterilization has become the preferred alternative to thermal sterilization.

As a nonthermal sterilization technology, cold plasma (CP) holds significant potential for application in fresh and heat-sensitive foods. CP, characterized by near-ambient temperature and exceptional chemical reactivity, generates energetic electrons through electric field acceleration. These electrons undergo inelastic collisions with gas molecules to form reactive oxygen/nitrogen species (ROS/RNS). These highly reactive transient species mediate diverse chemical interactions, notably in microbial inactivation where they selectively target bacterial cellular components: oxidizing lipid membranes via peroxidation, inducing DNA strand breaks through radical attack, and denaturing functional proteins by amino acid modification. This multi-mechanistic action enables efficient sterilization while preserving material compatibility, attributed to the non-thermal characteristics of CP [5,6]. The application of cold plasma in the sterilization and preservation of liquid food has been reported by relevant studies. Kumar et al. [7] found that 90 kV, 120 s high-voltage atmospheric cold plasma (HVACP) reduces Salmonella in coconut water by 1.30-log^10^ with minimal impact on physicochemical properties except for 84.35% loss of exogenous ascorbic acid. Aparajhitha et al. [8] reported atmospheric cold plasma cuts microbial count in sugar-rich Coconut Neera by ~80% with slight vitamin C reduction, inhibiting microbial growth in fruit and vegetable juices with little nutrient impact. Waghmare et al. [9] noted that cold plasma can prevent spoilage microbial growth in beverages, can inactivate endogenous enzymes, can retain physicochemical properties, requires a short treatment time, and has no obvious effect on quality parameters, being a promising sterilization technology to enhance beverage microbial safety while ensuring quality. Atmospheric pressure plasma jet (APPJ) introduces gas into a liquid to generate plasma inside it. APPJ technology has been applied in microbial inactivation [10], biomedicine [11], and food preservation [12]. The interaction between plasma and its reactive species with water in food is complex, particularly in liquid foods [13]. The use of APPJ in juices includes tomato juice [14], blueberry juice [15], maraschino cherry juice [16], and pomegranate juice [17], among others. Hou [15] demonstrated that APPJ not only deactivated Bacillus spp. with high efficacy but also better preserved the original color of blueberry juice. Wang [18] treated cherry juice with APPJ, noting a decrease in the stability of flavonols and anthocyanins while the stability of hydroxycinnamic acid increased. Currently, there are limited reports on the application of APPJ in coconut water, and the effects of treating APPJ on product quality remain unclear.

It has been found that heat treatment has a significant impact on the quality of coconut water and will destroy the physical and chemical quality and nutrient composition of coconut water, so it is important to choose CP, a non-thermal preservation technology. The application of CP in coconut water is mainly for sterilization and enzyme activity regulation. It can effectively kill microorganisms and reduce the activity of oxidase. Porto et al. [19] evaluated the effects of plasma and ozone treatment on the quality of coconut water. The CP treatment was evaluated as not changing the pH, total soluble solids, titratable acidity, and color, but reducing POD activity and changing its phenolic content. It was found that cold plasma treatment could effectively maintain and enhance the aroma of coconut water, and the treated coconut water retained key aroma compounds and increased the creaminess and popcorn flavor of coconut water [20,21]. Existing studies do not measure and evaluate the other physical and chemical qualities of coconut water after CP treatment and do not study the specific effects of CP treatment on its microorganisms during storage.

This study aims to optimize the processing time of APPJ treatment to maximize the preservation efficacy of coconut water. Specifically, it explores changes in key quality parameters during refrigerated storage, including color, turbidity, total soluble solids (TSS), enzyme activity, total phenolic content (TPC), and antioxidant capacity [assessed via ferric reducing antioxidant power (FRAP), DPPH, and ABTS assays]. Additionally, high-throughput sequencing targeting the V4 region of the 16S rRNA gene was employed to analyze microbial community structure, thereby evaluating the effects of CP treatment on coconut water microbiota. The findings of this study are expected to provide novel insights into coconut water preservation and quality control, while contributing to enhanced utilization efficiency of coconut resources.

## 2. Materials and Methods

### 2.1. Materials

Fresh coconut water was obtained from mature coconuts (8–9 months old) supplied by Hainan Fangye Agricultural Products Processing Co., Ltd., Chengmai, China. To ensure consistency in raw material quality, coconuts were selected based on strict criteria: uniform size (1.5–2.0 kg), intact husks free of visible damage, and clear liquid endosperm. The coconut water was extracted immediately under sterile conditions within 2 h of harvest and stored at (4 ± 1) °C until processing.

### 2.2. Methods

#### 2.2.1. Treatment of Coconut Water

**Figure 1:**
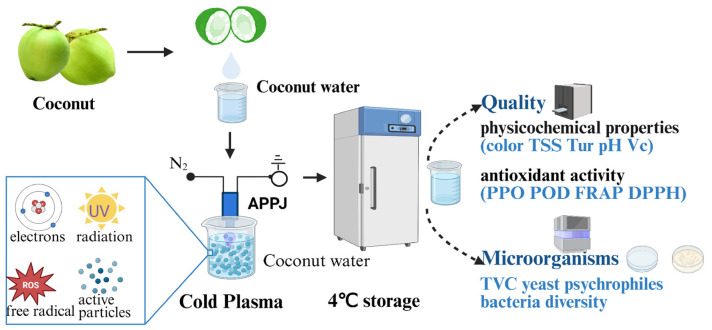
Experiment processing of coconut water.

Fresh coconut water was placed in a clean glass beaker and subjected to direct treatment using an atmospheric pressure plasma jet (APPJ) system (model PG-1000ZD, Nanjing Suman Plasma Technology Co., Ltd., Nanjing, China). Based on preliminary experimental results, the APPJ treatment parameters were set as follows: power at 800 W, nitrogen (N_2_, purity 99%) flow rate at 0.9 L/min, and treatment durations of 3 s, 6 s, and 12 s. Post treatment, the coconut water samples were stored at (4 ± 1) °C for 15 days, with sampling conducted every 3 days. The treatment process is shown in Figure 1.

#### 2.2.2. Color Measurement

We placed the coconut water into a clean glass container and used a spectrophotometer to measure the brightness value (L*), red-green value (a*), and yellow-blue value (b*) of the coconut water. The total color difference value ΔE* was calculated using the following formula [16,22]:

$$\Delta E*\;=\;\sqrt{{\left(L^{*}-L_{0}^{*}\right)}^{2}+{\left(a^{*}-a_{0}^{*}\right)}^{2}+{\left(b^{*}-b_{0}^{*}\right)}^{2}}$$

where L*, a*, b* represent the color values of the treated samples; *L*_0_*, a_0_*, b_0_* represent the color values of the untreated samples.

#### 2.2.3. Browning Index Measurement

The calculation formula for the Browning Index (*BI*) [23] is as follows:

$$BI=\frac{1000\times (x-0.31)}{0.172}\;\;\;\mathrm{where}\;x=\frac{a^{*}-1.75L^{*}}{1.75L^{*}+a^{*}-3.012b^{*}}$$

#### 2.2.4. Turbidity Test

The turbidity of the coconut water was measured using a UV spectrophotometer. The absorbance of the sample was measured at a wavelength of 610 nm, using distilled water for calibration [20]. The transmittance (*Tr*) and turbidity (*Tur*) were calculated with the following equations:

$$Tr=100\times 10^{-Ab}$$

Tur=100−Tr

#### 2.2.5. Chemical Quality Analysis

The pH value of the coconut water was measured with a pH meter (PHS-550, Hangzhou Luheng Biotechnology Co., Ltd., Hangzhou, China).

The total soluble solids (TSS) were measured with a hand refractometer with results expressed as a percentage.

The method for measuring Titratable acidity (TA) was modified from Ceballos [17,24]. TA was assessed by titrating a known volume of coconut water with 0.1 mol/L sodium hydroxide until a persistent pink endpoint was achieved, with results also expressed as a percentage.

The total sugar content in coconut water was measured using the phenol–sulfuric acid method, as described by Wang [18]. The results were expressed in grams of glucose equivalents per 100 mL (g GE/100 mL).

The total reducing sugar content was estimated using the dinitrosalicylic acid (DNSA) method, based on Liao [10]. A standard curve was prepared using glucose as the reference. The results were expressed in grams of glucose equivalents per 100 mL (g GE/100 mL).

TPC was measured via the Folin–Ciocalteu method with gallic acid as the reference compound, following Prithviraj [25]. The absorbance of the sample solution was measured at 765 nm using a UV spectrophotometer. TPC was expressed in milligrams of gallic acid equivalents per 100 mL of coconut water (mg GAE/100 mL).

Total ascorbic acid content (TAA) was determined using a TAA assay kit sourced from Suzhou Grace Biotechnology Co., Ltd., Suzhou, China, and a microplate method was utilized.

#### 2.2.6. PPO and POD Enzyme Activity Measurement

The activities of polyphenol oxidase (PPO) and peroxidase (POD) were measured using assay kits (Suzhou Grace Biotechnology Co., Ltd., Suzhou, China), both employing the microplate method.

#### 2.2.7. Antioxidant Capacity Measurement

The scavenging rates of DPPH and ABTS^+^ were determined following the methods described by Ceballos and Elcik [26,27]. For the DPPH assay, 0.25 mL of coconut water was mixed with 2 mL of 0.1 mmol/L DPPH solution, and the mixture was stored in the dark for 30 min. Absorbance was then measured at 517 nm. For the ABTS assay, an ABTS working solution was first prepared by mixing 2.6 mM potassium persulfate with 7.4 mM ABTS salt in ethanol, adjusted to an absorbance of 0.7 ± 0.02 at 734 nm. Subsequently, 0.1 mL of the sample was mixed with 1.9 mL of the ABTS working solution, incubated in the dark for 30 min, and absorbance was measured at 734 nm.

#### 2.2.8. Microbiology Amount Determination

One milliliter of coconut water was mixed with 9 mL sterile saline and diluted to the appropriate concentration using the gradient dilution method. Aliquots of the appropriately diluted solutions were spread onto agar media, which were then incubated under specific conditions: total viable colonies were cultured at 37 °C for 24 h; psychrophilic bacteria were cultured at 4 °C for 12 days; lactic acid bacteria and yeast were incubated at 37 °C for 24 h and 25 °C for 72 h, respectively [28]. After incubation, colonies were enumerated, and the results were expressed as log colony-forming units per milliliter (lg CFU/mL).

#### 2.2.9. Bacteria Diversity Analysis

High-throughput sequencing technology was employed to analyze changes in bacterial diversity [29]. Genomic DNA was extracted from the bacterial community in coconut water using an ALFA Soil DNA Extraction Kit (Ark Biosafety Technology (Guangzhou) Co., Ltd., Guangzhou, China), followed by the quantification of DNA concentration and the assessment of integrity. PCR amplification targeting the V3-V4 variable regions of the 16S rRNA gene was performed using the primer v3v4-1. The PCR conditions were as follows: pre-denaturation at 94 °C for 5 min, followed by 30 cycles of denaturation at 94 °C for 30 s, annealing at 52 °C for 30 s, and a final extension at 72 °C for 10 min. High-throughput sequencing was conducted on the Illumina NovaSeq 6000 platform in PE250 mode, yielding a read length of approximately 469 bp [30].

Library construction was carried out following the standard protocol of the ALFA-SEQ DNA Library Prep Kit. Library quality control included fragment size evaluation using a Qsep400 high-throughput nucleic acid analyzer (Hangzhou Houze Biotechnology Co., Ltd., Hangzhou, China) and concentration determination via Qubit 4.0 (Thermo Fisher Scientific, Waltham, MA, USA). Based on 16S rRNA gene sequencing data, changes in the microbial community structure of coconut water treated with cold plasma (CP) for 12 s during storage were analyzed.

### 2.3. Statistics Analysis

All experiments were conducted three times, with data presented as mean ± standard deviation. Data calculations were performed using Excel 2010, graphs were drawn using Origin 2021 (Originlab, Northampton, MA, USA), and a one-way analysis of variance (ANOVA) was conducted using SPSS Statistics 25 (SPSS Inc., Chicago, IL, USA).

## 3. Results and Discussion

### 3.1. Quality

#### 3.1.1. Color and Browning Index

Samples treated with different CP durations showed no significant differences in ΔE* values (Table 1), indicating that the CP treatment does not affect the color of coconut water and is conducive to preserving its sensory quality. This is consistent with a similar study on sugarcane juice, where dielectric barrier discharge (DBD) plasma treatment resulted in a color difference (ΔE*) of <0.5, reflecting only a slight color change [31].

The browning index (*BI*) is a key indicator reflecting the browning degree of beverages, which results from color changes caused by oxidative reactions of phenolic compounds in food upon exposure to oxygen. In this study, the BI of coconut water showed an increasing trend with extended CP treatment time (Table 2). Additionally, BI increased with prolonged storage, with no significant differences observed among different treatment groups. This storage-related *BI* increase may enhance the oxidative discoloration of coconut water due to increased dissolved oxygen over time. However, CP treatment could delay browning to some extent by inhibiting the activities of endogenous oxidative enzymes (e.g., peroxidase and polyphenol oxidase) [32], thereby contributing to the preservation of sensory quality. Consistent results have been reported in CP-treated orange juice [28], apple juice [30], and carrot juice [24]. Similarly, atmospheric cold plasma (ACP) treatment was found to have no adverse effects on the pH, total soluble solids, titratable acidity, or color of coconut water; high-frequency ACP treatment minimized the residual activity of POD and caused only slight changes in phenolic compound content. These findings indicate that plasma treatment can inhibit browning-related enzyme activities in coconut water to a certain extent, which is beneficial for maintaining color stability and reducing browning [22].

#### 3.1.2. pH

**Figure 2:**
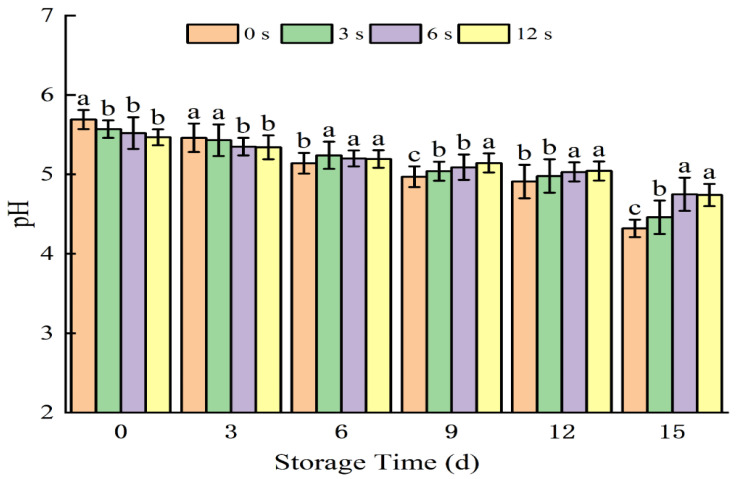
pH changes in cold plasma-treated coconut water during storage. Note: letters indicate the differences between different treatment groups.

The pH of coconut water decreased after CP treatment, with a significant reduction (*p* < 0.05) observed as the treatment time was extended (Figure 2). This phenomenon can be attributed to the production of acidic components by nitrogen-generated CP, which causes a rapid short-term decline in the pH of coconut water [33]. After 6 days of storage, the pH values of CP-treated samples became higher than those of the untreated control. Over prolonged storage, microbial growth and reproduction in coconut water typically consume sugars and produce metabolites such as lactic acid and acetic acid, leading to a gradual pH decrease. However, CP treatment inhibited the growth rate of microorganisms in coconut water, resulting in a slower pH decline compared to the control group [34].

#### 3.1.3. Turbidity

**Figure 3:**
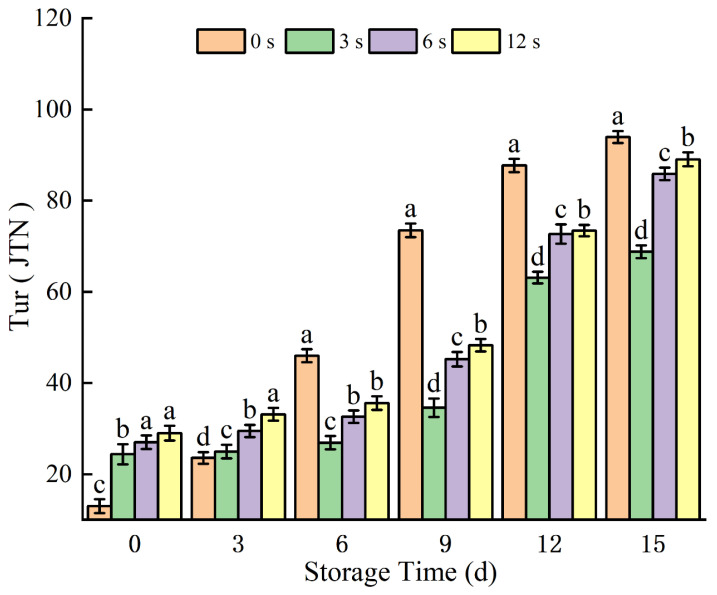
Turbidity changes in cold plasma-treated coconut water during storage. Note: letters indicate the differences between different treatment groups.

Turbidity was used as an indicator to evaluate quality changes in coconut water during storage. As shown in Figure 3, turbidity value increased in all groups throughout the cold storage period, reflecting the gradual accumulation or suspension of certain components in coconut water over time. This storage-related turbidity increase may be attributed to two main factors: The first is microbial growth decomposing macromolecular nutrients, which increases the content of suspended particles [4]. The second is oxidative reactions catalyzed by polyphenol oxidase (PPO) and peroxidase (POD) in coconut water upon exposure to oxygen, leading to a transition from clarity to turbidity [35]. Notably, after 3 days of refrigeration, the turbidity values of CP-treated samples were lower than those of the control (CK) group, indicating that CP treatment inhibited microbial growth and enzymatic browning, thereby enhancing system stability despite the overall gradual increase in turbidity. This aligns with previous findings that nitrogen-based cold plasma (N_2_-CP) can effectively inhibit microorganisms and pathogens in aqueous samples [36], which helps maintain sample clarity. Similar trends have been observed in other liquid systems: Rajashri [4] reported that plasma-treated guar gum increased the stability of orange juice, reducing sedimentation and viscosity while increasing turbidity; conversely, Kuma [7] found that CP treatment significantly reduced the turbidity of kiwi juice, and Vukić [37] noted increased turbidity in a mixture of orange juice and carrot juice after DBD plasma treatment. Among the tested groups, samples treated for 3 s showed the lowest turbidity values after day 3, indicating that CP-3 s treatment can maximize the uniformity of coconut water.

#### 3.1.4. Contents of TSS and TA

**Figure 4:**
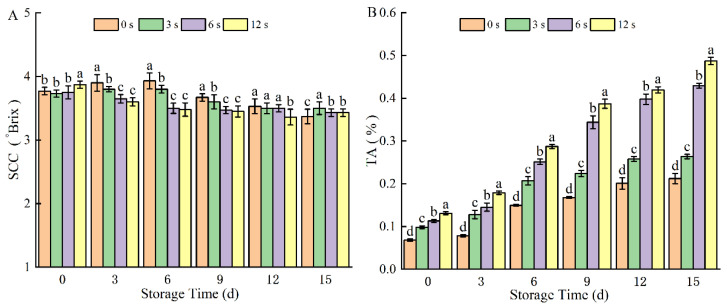
Total soluble solids (**A**) and total titratable acidity (**B**) of cold plasma-treated coconut water during storage. Note: letters indicate the differences between different treatment groups.

After 6 s and 12 s of CP treatment, TSS content of coconut water showed a slight decrease compared to the control group (Figure 4A). This minor decline can be attributed to the inhibitory effect of CP on microbial growth, which slows down sugar degradation processes, resulting in a relatively small reduction in TSS during storage. Consistent with these findings, Tan et al. [38] observed negligible changes in TSS when treating coconut water with DBD plasma. Similarly, Silva et al. [39] reported that plasma treatment had no significant impact on the pH or TSS content of fruit juices, further supporting the stability of TSS in plasma-treated liquid systems.

As the storage time extended, the TA values were increased across all sample groups (Figure 4B). This upward trend was attributed to microbial fermentation processes during storage: the extensive growth and proliferation of microorganisms (e.g., yeasts and lactic acid bacteria) in coconut water led to the consumption of sugars and the production of organic acids such as lactic acid and acetic acid, which collectively contributed to the increase in TA [40]. Furthermore, CP treatment induced a decrease in pH, creating an acidic environment in coconut water. This environment likely preserved organic acids or slowed their decomposition and consumption rates, potentially contributing to the increase in TA. Consistent with this, Silva [39] observed increased acidity in probiotic orange and apple juices following CP treatment. Moreover, after 12 s CP treatment, the TA value of coconut water was significantly increased, and this trend continued with extended CP treatment duration. This phenomenon suggested that CP facilitates the acidification of coconut water, likely attributed to the active substances of nitrogen oxides (NO_X_) in N_2_-CP, resulting in decreased pH and elevated TA in coconut water. Similar studies have reported that DBD plasma treatment with a treatment power of 39 W and a processing time of 120 s resulted in an increase in the TA of TNM. The increase in acidity may be due to OH during plasma treatment [41].

Reactive particles generated by plasma can oxidize sugars to produce acids; simultaneously, proteolysis releases amino acids, increasing the number of acidic carboxyl groups (-COOH) in the system [39]. This aligns with previous reports that CP treatment reduces the pH of beverages such as orange juice [42] and apple juice [32], a phenomenon attributed to the formation of plasma-derived chemical species (e.g., hydrogen peroxide), which contribute to acidity development in the beverages. During storage, the continued decrease in pH may be linked to the accumulation of reactive species and nitrogen-containing acids generated by plasma. CP treatment can either reduce or increase sugar content in fruits and juices, with the underlying mechanism potentially involving metabolic shifts in cellular defense responses. Specifically, CP exposure may induce a stress response in fruits, triggering the utilization of sugars for phenolic compound biosynthesis. This process would result in decreased sugar content alongside increased phenolic compounds [43].

#### 3.1.5. Activities of PPO and POD

**Figure 5:**
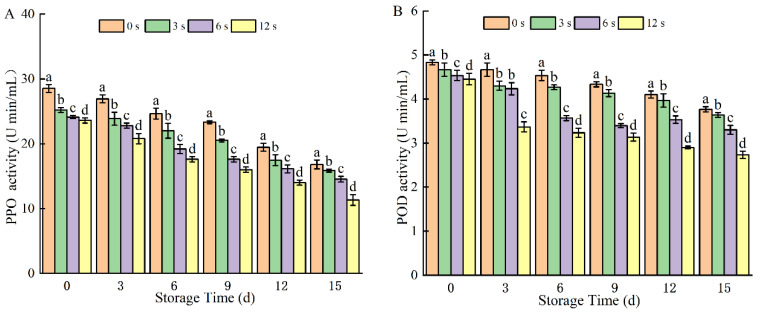
Polyphenol oxidase activity (**A**) and peroxidase activity (**B**) of cold plasma-treated coconut water during storage. Note: letters indicate the differences between different treatment groups.

PPO and POD are key oxidative enzymes in coconut water, playing critical roles in enzymatic browning; thus, reducing their activities directly impacts the browning reaction process. As shown in Figure 5, the activities of PPO and POD exhibited a decreasing trend across all groups. Specifically, PPO and POD activities in coconut water decreased with extended CP treatment time: after 15 days of storage, 12 s CP treatment resulted in a 32.56% reduction in PPO activity compared to the control. The underlying mechanism of enzyme inactivation may involve multiple pathways: reactive species generated during CP treatment can attack amino acid residues in PPO and POD, disrupting their secondary and tertiary structures. Additionally, ultraviolet (UV) radiation produced in the plasma can induce protein structural changes, leading to enzyme deactivation [4]. Structural analyses have revealed that CP-induced enzyme inactivation is associated with alterations in α-helix and β-sheet conformations. For instance, low-frequency plasma jets have been reported to induce chemical modifications in 14 amino acids, with amino acid residue modifications and bond cleavage potentially triggering structural changes in PPO and subsequent loss of activity [13,44]. Dong et al. [42] further demonstrated that plasma deactivates PPO by increasing β-sheet content and decreasing α-helix content, which induces oxidative degradation, carbonylation, and loss of catalytic activity in PPO. Similarly, Sreelakshmi [45] also found that plasma treatment reduced PPO and POD activity in apples during storage. A comparative study of non-thermal technologies (ozone vs. cold plasma) showed that CP treatment resulted in the lowest residual POD activity in coconut water [19], highlighting its effectiveness in reducing POD activity. Combined with color and phenolic substances, which reflected the important potential of cold plasma in delaying the oxidative browning of coconut water. In this study, activities of PPO and POD enzymes decreased with the increase in treatment time and were still significantly lower than those of the control group at the end of storage.

#### 3.1.6. Contents of Total Sugar and Reducing Sugar

**Figure 6:**
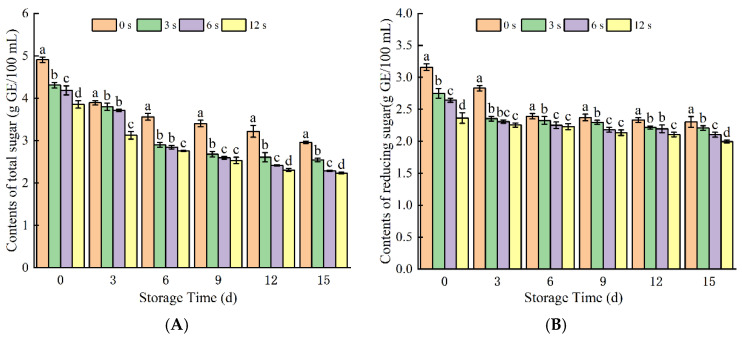
Total sugar (**A**) and reducing sugar (**B**) content of cold plasma-treated coconut water during storage. Note: letters indicate the differences between different treatment groups.

CP treatment significantly affected the contents of both total sugar and reducing sugar in coconut water (Figure 6), with the treated group exhibiting notably lower levels compared to the control group. With prolonged storage time, there was a decreasing trend in reducing sugar across all groups, although the reduction was not significant. For the active species in CP were reacted with the sugar in coconut water, which disrupts the tissue matrix and releases nutrients during the treatment. It has been reported that DBD plasma treatment of coconut water induces cell–matrix disruption and sugar consumption, even with relatively short treatment durations [22,34]. In the present study, no significant differences in relevant parameters were observed among the treatment groups during the storage period from 6 to 15 d.

Notably, the total reducing sugar content decreased by 39.82% after 12 s of CP treatment, which might be attributed to sugar consumption by microbial growth and proliferation. Kalaivendan [44] observed a reduction in reducing sugar of orange juice, after CP treatment, possibly due to an increase in sucrose and a decrease in fructose, while glucose remained unchanged, resulting in a decrease in reducing sugar content [36]. The degradation of glucose and fructose was attributed to the formation of reactive plasma species, such as hydroxyl radicals, atomic oxygen, or singlet oxygen. These radicals react with the sugars and produce acidic compounds [36]. Additionally, the consumption of reducing sugar in coconut water during storage may also result from fermentation processes. In summary, the observed reduction in sugar content can be attributed to two main factors: the microbial utilization of sugars for metabolic activities and the involvement of sugars in enzymatic or non-enzymatic browning reactions.

#### 3.1.7. Contents of TPC and TAA Content

**Figure 7:**
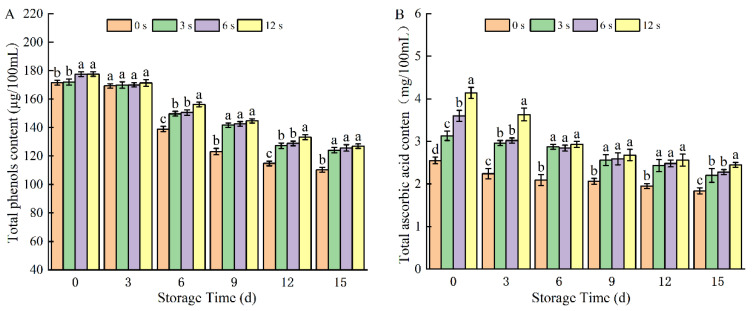
Total phenol content (**A**) and total ascorbic acid content (**B**) of cold plasma-treated coconut water during storage. Note: letters indicate the differences between different treatment groups.

As the storage time increased, both the control and treated groups experienced a decrease in TPC and TAA, while CP treatment boosted the phenolic content in coconut water (Figure 7). Prolonging CP treatment increased the contents of both TPC and TAA in coconut water, which was attributed to the decreased activities of PPO and POD. On day 15, the treated group exhibited significantly higher levels of TPC and TAA compared to the control group. CP treatment for 12 s effectively counteracted the decline in TPC and TAA during storage. Previous studies have reported that nitric oxide (NO) generated during CP treatment can enhance the activity of dehydroascorbate reductase, thereby promoting the synthesis of ascorbic acid [46]. However, as storage time increased, the loss of ascorbic acid intensified, which may be associated with the continuous oxidation of ascorbic acid by reactive oxygen species (ROS) generated by residual plasma effects during storage. Similar trends have been observed in other fruit juice systems: plasma treatment increased phenolic content in tropical fruit juices, while DBD plasma treatment induced a marginal increase in phenolic compounds in strawberries. Porto [19] discovered that treating with CP inhibited PPO and POD activity, thereby reducing phenolic oxidation and improving browning. Following treatment, coconut water experienced an increase in TPC and flavonoids, enhancing its antioxidant capacity. Nasri [46] noted that plasma treatment induced the degradation of aromatic rings in carrot juice, releasing more phenolic compounds and thereby augmenting the TPC. The increase in TAA observed in this study may be attributed to the generation of free radicals induced by plasma treatment, which in turn leads to a reduction in ascorbic acid content. Additionally, Sarangapani et al. [45] reported that atmospheric pressure CP treatment enhanced the TAA of blueberries.

#### 3.1.8. Antioxidant Capacity

**Figure 8:**
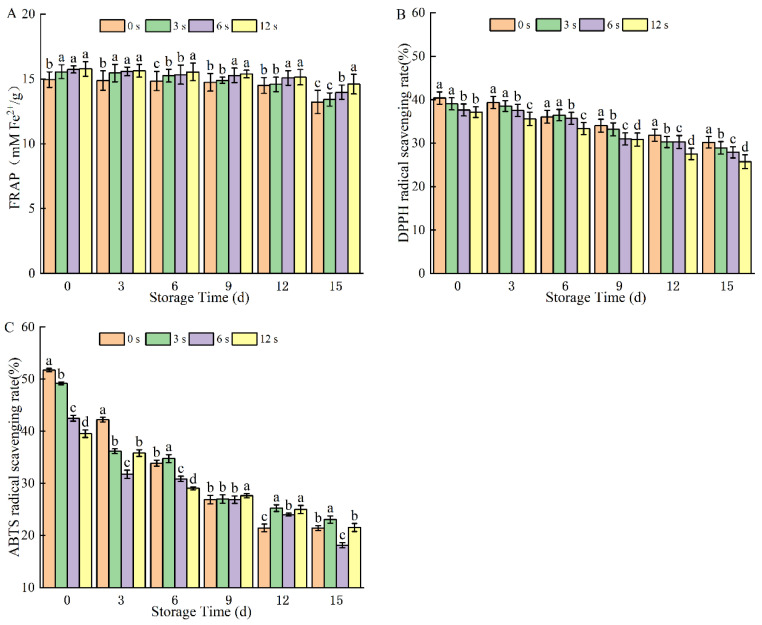
Iron restoration antioxidant capability (**A**), DPPH free radical clearing rate (**B**), and ABTS^+^ free radical clearing rate (**C**) of cold plasma-treated coconut water during storage. Note: letters indicate the differences between different treatment groups.

The total antioxidant capacity of coconut water is related to its food characteristics, phenolic content, and other factors, and FRAP is an important indicator for evaluating the overall antioxidant capacity of coconut water. The FRAP increased after plasma treatment, and it increased by 5.48% after 12 s of treatment (Figure 8). It increased gradually over the storage period, which is consistent with the observed trends in TAA and oxidative enzyme activity. CP treatment has been proven to boost the antioxidant capacity of fresh-cut pears [2], cashew apple juice [47], and chili peppers [48]. Increasing the excitation frequency and treatment duration of CP can diminish the antioxidant capacity of chokeberry juice [49]. These studies collectively demonstrate that CP treatment exerts a preservative effect on the antioxidant properties of fruit and vegetable juices. Specifically, under appropriate treatment conditions, CP treatment not only reduces microbial counts but also maintains or even enhances antioxidant capacity, thereby preserving the antioxidant efficacy of the juices.

The DPPH and ABTS^+^ free radical scavenging rates of CP-treated coconut water exhibited a declining trend during refrigerated storage. The DPPH and ABTS^+^ scavenging rates of samples treated for 12 s increased to 37.15% and 39.51%, respectively. As reactive species generated by CP attack enzymes in coconut water to reduce their activity, the production of free radicals is inhibited, thereby relatively enhancing antioxidant capacity. Notably, the decline rate of free radical scavenging rates in CP-treated samples was significantly lower than that in untreated samples. However, as CP treatment time extended, prolonged contact between coconut water and plasma-derived reactive species induced oxidative stress, which contributed to a reduction in free radical scavenging ability. This trend was further exacerbated during refrigerated storage: prolonged exposure to oxygen promoted oxidative reactions in coconut water over time, leading to a gradual decrease in DPPH and ABTS^+^ free radical scavenging rates despite the initial antioxidant-enhancing effect of CP. Similar reports found that dielectric barrier discharge cold plasma treated tender coconut water, DPPH scavenging activity decreases with plasma treatment time and voltage [41]. Combining TPC and TAA mentioned above, CP treatment can maintain the antioxidant capacity of coconut water during refrigeration by delaying the loss of antioxidants.

### 3.2. Microorganism Counts

**Figure 9:**
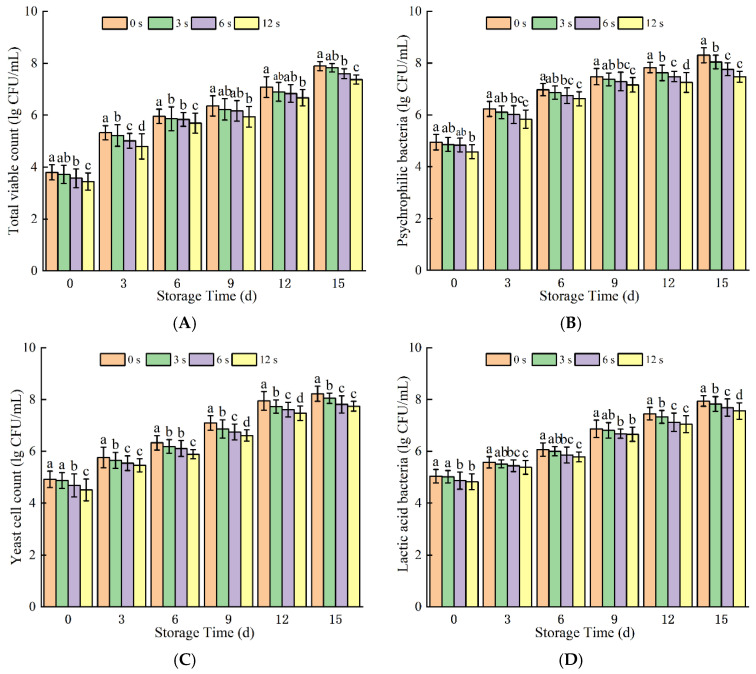
Total viable count (**A**), psychrophilic (**B**), yeast (**C**), and lactic acid bacteria (**D**) of cold plasma-treated coconut water during storage. Note: letters indicate the differences between different treatment groups.

The main changes in bacteria amount in the coconut water are shown in Figure 9. The total viable count (TVC) decreased as the treatment time increased and increased gradually during the storage period. After treatment for 3 s, 6 s, and 12 s, the total aerobic bacterial count in coconut water decreased from the initial 3.80 lg CFU/mL to 3.73 lg CFU/mL, 3.57 lg CFU/mL, and 3.44 lg CFU/mL, respectively. Specifically, 12 s CP treatment resulted in a 0.30 lg CFU/mL reduction in the total yeast count, while the total lactic acid bacteria count decreased from 5.04 lg CFU/mL to 4.92 lg CFU/mL. Notably, at 15 days of storage, the total viable count (TVC) in the 12 s CP treatment group was significantly lower than that in other groups (*p* < 0.05). Psychrophilic bacteria and yeast exhibited a similar growth trend to total viable count (TVC) during storage, with yeast counts remaining significantly lower than TVC throughout the period. In contrast, the total lactic acid bacteria (LAB) count in coconut water gradually increased with prolonged storage. This LAB proliferation may be attributed to two factors: first, the decrease in pH induced by CP treatment created an acidic environment favorable for LAB growth; second, as facultative anaerobes, LAB may have benefited from reduced oxygen levels during refrigerated storage, which further promoted their growth and reproduction.

During the treatment process, reactive species interact with components in coconut water, where reactive oxygen and nitrogen species (RONS) induce stress-mediated microbial cell death. As treatment time extends, the increased production of reactive components in coconut water and prolonged contact between plasma-derived active substances and microorganisms synergistically enhance antimicrobial effects: these reactive species disrupt microbial cell membranes, exacerbate oxidative damage to intracellular macromolecules (e.g., nucleic acids and proteins), and ultimately inhibit normal microbial growth and reproduction [50]. As the attacking effect of bacteria cells accumulated, it ultimately led to the loss of microbial viability or death. For microbial load in coconut water, Kumar et al. [7] reported that HVACP treatment at 90 kV for 120 s reduced Salmonella in coconut water by 1.30-log^10^. Similar studies on fruit beverages also show significant microbial reduction. However, coconut water was treated with nitrogen-based cold plasma, and it was observed that it had a bactericidal effect, but compared with air-source cold plasma, air-CAP had better inactivation ability than nitrogen-CAP, and the damage to cell membranes by air-CAP may be more serious than that of nitrogen-CAP, which can lead to the fragmentation of biological macromolecules [51]. Similarly, the CP treatment of coconut water with air as the working gas significantly reduced the TVC [52,53] and inhibited the TVC amounts in sugarcane juice [31]. CP with oxygen as the working gas, effectively reduced its psychrophilic bacteria count in freeze-dried bovine colostrum [50]. Similarly, (CP) treatment has been shown to reduce lactic acid bacteria (LAB) counts in radish kimchi. For orange juice, both direct and indirect treatment with HVACP effectively reduces populations of Salmonella enterica serovar Typhimurium (S. enterica): 120 s of indirect HVACP treatment followed by 24 h storage resulted in a 2.2-log reduction in air-packaged samples and a 3.8-log reduction in MA65-packaged samples [54]. Additionally, HVACP using air as the excitation gas exhibits a positive antimicrobial effect against S. enterica, achieving a 2.2-log reduction in bacterial counts [54]. In the present study, the relatively low reduction in total colony counts may be attributed to differences in treatment parameters, such as working gas type and power output. Specifically, nitrogen-based CP has been reported to exhibit slightly weaker sterilization efficacy compared to air-based CP, which could further explain the moderate antimicrobial effect observed herein.

### 3.3. Correlation Relationship Between Physicochemical Quality and Microorganism

**Figure 10:**
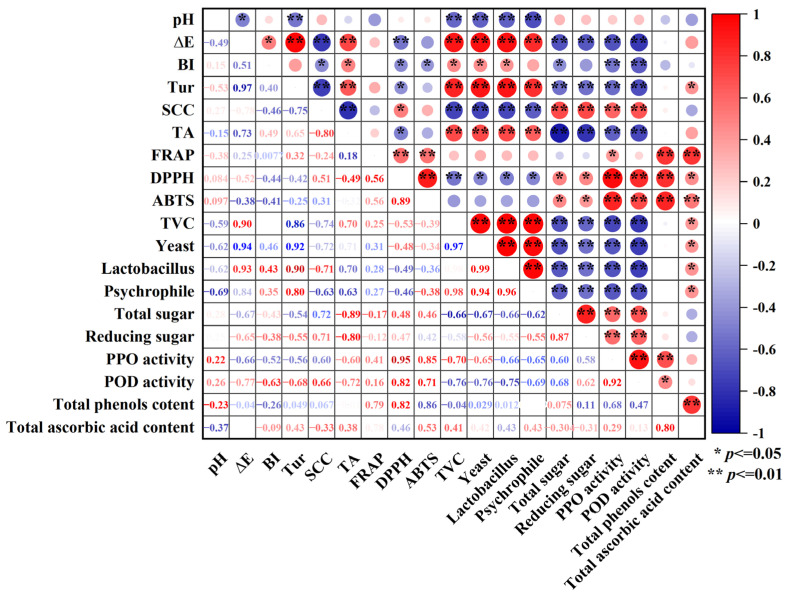
Correlation relationship between physiochemical qualities and microorganisms of cold plasma-treated coconut water during storage.

The correlation relationship among the 19 indicators measured was analyzed with the Pearson correlation during the storage period (Figure 10) to determine the key indicators for good preservation effects of coconut water. As shown in the figure, the ∆E and Tur are positively correlated with the microbial indicators TVC, Yeast, Lactobacillus, and Psychrophile. The higher the values of ∆E and Tur were, the more severe the microbial contamination and the lower the sensory quality were, which is consistent with the increase in color changes, turbidity, and microorganisms. The ∆E and Tur are extremely significantly positively correlated with the numbers of TVC, Yeast, Lactobacillus, and Psychrophile (*p* < 0.01). Similarly, pH is significantly negatively correlated with the numbers of TVC, Yeast, Lactobacillus, and Psychrophile (*p* < 0.05). This phenomenon may be attributed to the decrease in coconut water pH, which enhances the bioavailability of certain nutrients to specific microorganisms, thereby promoting microbial proliferation. Concurrently, acidic metabolites produced during microbial metabolism further lower the pH of coconut water, forming a feedback loop. The observed increase in TA may stem from microbial degradation of sugars into organic acids such as lactic acid, which is accompanied by reductions in total sugar and reducing sugar contents. Additionally, a highly significant positive correlation (*p* < 0.01) was observed between free radical scavenging rates (DPPH and ABTS^+^) and TPC in this study. The higher the free radical scavenging rate of coconut water, the higher the total phenolic content and the stronger the antioxidant capacity.

### 3.4. Bacteria Diversity

#### 3.4.1. Sequencing Analysis

Based on the aforementioned microbial data, coconut water treated for 12 s was selected for microbial diversity analysis. The changes in microbial alpha diversity of coconut water during storage were shown in Table 3. The microbial community diversity indices, including Richness, Chao 1, Shannon, and Simpson, exhibited a trend of first decreasing and then increasing during storage. This dynamic change indicates that the richness and diversity of microbial communities in coconut water underwent continuous fluctuations over the storage period, which is consistent with the observed variations in the number of operational taxonomic units (OTUs).

The sequencing data were considered to cover most of the microbial diversity of the samples. As shown in Table 4, the valid sequences of coconut water were analyzed and annotated by OTUs clustering according to 97% similarity. The number of OTUs exhibited a dynamic trend of initial increase, subsequent decrease, and final re-increase during storage. This pattern can be interpreted as follows: in the early stage of storage, the surviving microorganisms in CP-treated coconut water gradually regained metabolic activity, undergoing growth and reproduction, which contributed to the initial rise in OTU numbers. During the middle stage of storage, as the storage environment stabilized, intense interspecific competition occurred among microbial populations in coconut water. This competitive pressure likely led to the reduction in OTU numbers, with no significant fluctuations in microbial diversity observed during this period.

**Figure 11:**
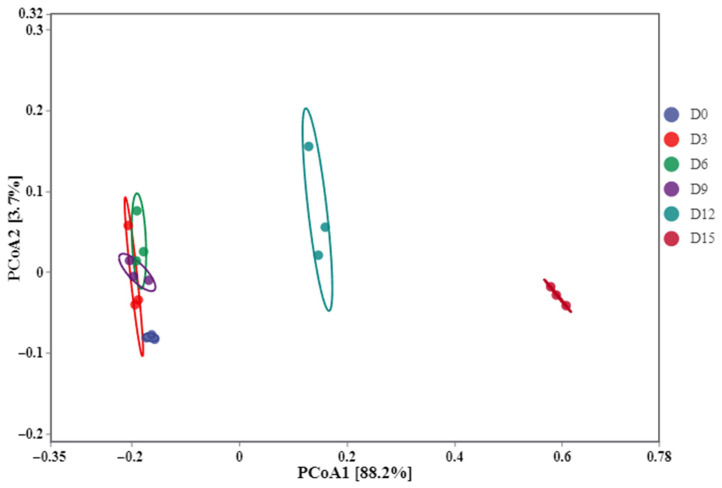
Principal Coordinate Analysis (PCoA) of cold plasma-treated coconut water during storage.

Principal Coordinate Analysis (PCoA) was employed to analyze the differences in the microbial community structure of coconut water during refrigeration storage (Figure 11). The first principal component (PC1) accounted for 88.2% of the total variance, indicating that it was the most influential factor driving changes in microbial community structure. In contrast, the second principal component (PC2) contributed only 3.7% to the variance. Notably, samples collected at days 0 (D0), 3 (D3), 6 (D6), and 9 (D9) of storage clustered closely in the PCoA plot, with minimal spatial distance between them. This clustering pattern indicates that the microbial community compositions among these samples did not differ significantly, with their microbial species profiles remaining highly consistent during the early to mid-storage stages. During the first 9 days, CP treatment damaged parts of the microorganisms and limited their growth and reproducing ability. The D12 and D15 samples were relatively independent and distinct, indicating that the changes in microbial communities in coconut water during this period were significant.

#### 3.4.2. Phylum Level Classification Analysis

**Figure 12:**
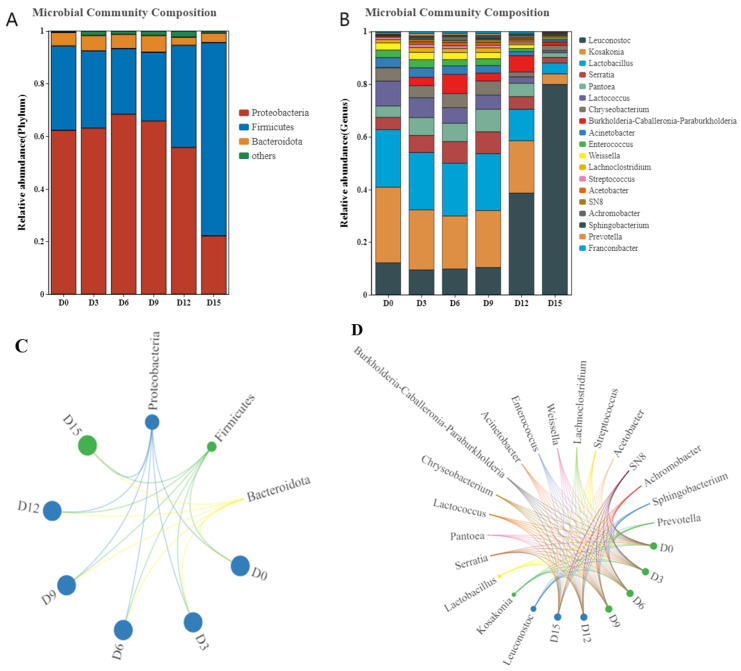
The diversity distribution of bacterial communities at the phylum (**A**,**C**) and genus (**B**,**D**) levels in cold plasma-treated coconut water during storage.

The diversity distribution of bacterial communities at the phylum and genus levels in coconut water is shown in Figure 12. Proteobacteria, Firmicutes, and Bacteroidota were the dominant bacterial phyla in coconut water with high abundance. Proteobacteria had the highest abundance ratio and was the dominant phylum until 12 d, after which the proportion of Firmicutes exceeded 60%, surpassing the content of Proteobacteria. Therefore, CP treatment exerted distinct effects on the dominant bacterial phyla in coconut water during different storage stages. Within the first 6 days of storage, CP treatment led to an increase in the relative abundance of Proteobacteria while reducing the abundances of Firmicutes and Bacteroidota. In contrast, between days 9 and 15, the trend reversed: CP treatment resulted in decreased abundances of Proteobacteria and Bacteroidota, accompanied by an increase in Firmicutes.

As shown in Figure 12B,D, the main genera in coconut water are *Leuconostoc*, *Kosakonia*, *Lactobacillus*, *Serratia*, *Pantoea*, and *Lactococcus*. After 15 days of storage, *Leuconostoc* emerged as the most abundant genus, whereas *Serratia* exhibited the highest abundance between days 6 and 9, and *Pantoea* dominated in samples collected on day 9. In contrast, the relative abundances of *Kosakonia*, *Lactobacillus*, and *Lactococcus* showed a downward trend throughout refrigerated storage. Proportions of *Kosakonia*, *Lactobacillus*, and *Lactococcus* displayed a downward trend during the refrigeration process. The underlying mechanism for these shifts may involve the antimicrobial effects of CP: reactive species generated during CP treatment can attack bacterial cells, damaging cell walls to increase membrane permeability. This process ultimately leads to cell membrane rupture, followed by damage to organelles, proteins, and nucleic acids, culminating in microbial cell death [55,56]. Specifically, the abundance of *Kosakonia* was slightly reduced from 0 to 9 days, and *Lactobacillus* showed a similar trend. *Lactobacillus* was one of the dominant genera, which is a beneficial bacterium and conducive to maintaining the good quality of coconut water. During 12 to 15 days, the *Leuconostoc* increased and became dominant, exceeding the *Lactobacillus*.

**Figure 13:**
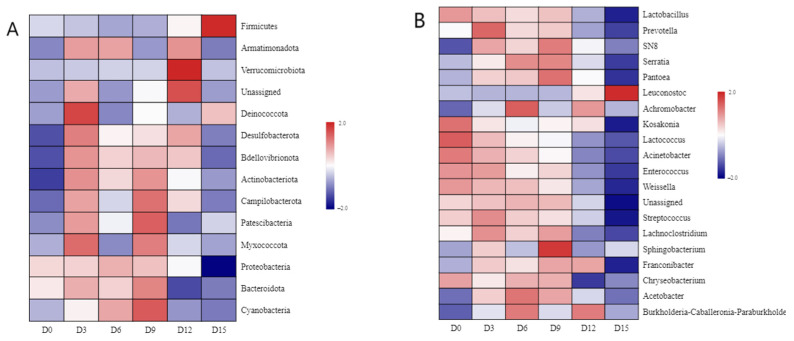
Heat map of the distribution of species abundance at phylum (**A**) and genus (**B**) levels of OTU in cold plasma-treated coconut water during storage. (The figure notes indicate that the horizontal represents the sample and the vertical is the species (phylum/genus). The abundance difference between the groups is compared by the square color. The redder the color of square, the higher the abundance of the phylum/genus is between the samples.)

Based on the heatmap analysis (Figure 13), the top 15 phyla were Firmicutes, Verucomicrobiota, and Deincoccota in coconut water. The main genera were *Leuconostoc*, *Lactobacillus*, and *Sphingobacterium*. No significant differences in genus-level relative abundances were observed among samples from day 0 to day 6. In the day 9 (D9) group, *Sphingobacterium* emerged as the dominant genus; this bacterium is capable of degrading aromatic compounds in coconut water and producing extracellular polymeric substances, which may induce sensory quality changes such as flocculent sedimentation and flavor deterioration. *Leuconostoc* was the dominant genus in the day 15 (D15) group. Notably, *Leuconostoc* can synthesize various antimicrobial substances, including lactic acid and bacteriocins, which inhibit the growth of pathogenic and spoilage microorganisms [57]. Its acid tolerance and strong capacity for sugar fermentation—accompanied by acid and gas production—enable it to effectively outcompete other genera. These physiological characteristics collectively contributed to the dominance of *Leuconostoc* in the D15 sample.

**Figure 14:**
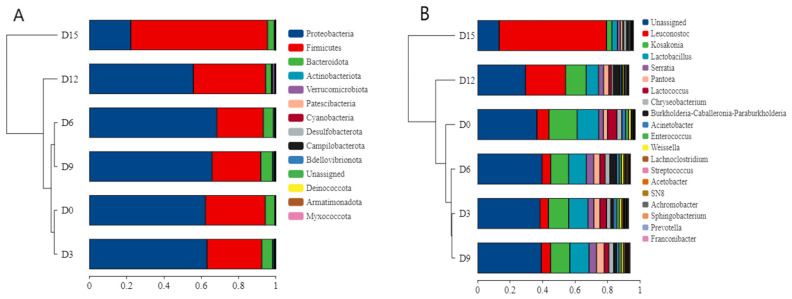
Sample hierarchical clustering tree of species abundance at phylum (**A**) and genus (**B**) levels of OTU in cold plasma-treated coconut water during storage.

To study the similarity or correlation of community structure in coconut water during storage, cluster analysis was performed on the sample community distance matrix. At the phylum level (Figure 14A), the hierarchical clustering results between the D0 and D3 groups were similar, indicating no significant changes in the microbial community structure. The community structure of the D15 sample was significantly different from the other groups. Similarly, at the genus level (Figure 14B), the D15 sample was quite different, with significant changes in *Leuconostoc*, *Kosakonia*, and *Lactobacillus*.

**Figure 15:**
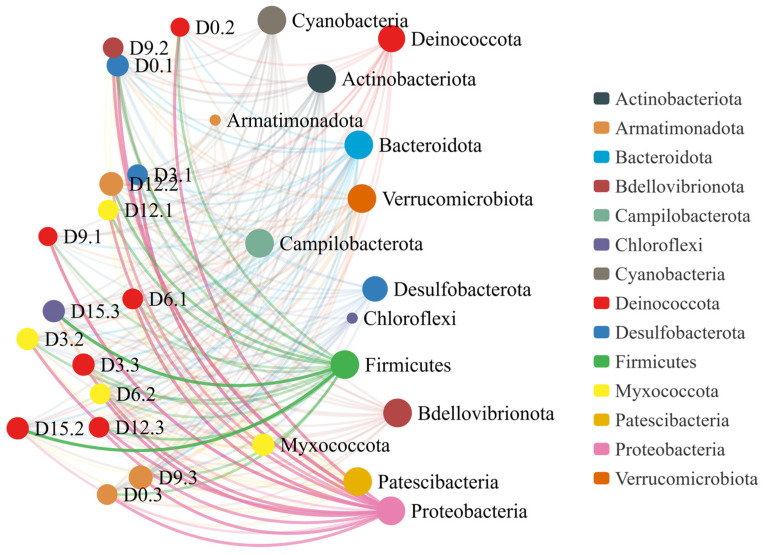
Collinear network distribution of cold plasma-treated coconut water during storage (gate level of OTU).

The co-linearity network characteristics of microbial communities at the phylum level during storage are shown in Figure 15. The thickness of the connecting lines indicates the strength of the correlation, and the same node color represents the same phylum. The relationships among different bacterial phyla were significant in coconut water during storage. In our study, 15 notes had high connectivity in the network diagram. The highest connectivity appeared between Proteobacteria and Firmicutes, which accounted for the largest proportion of all nodes. Other phyla only existed during certain storage periods, showing low connectivity and smaller connection points.

## 4. Conclusions

CP treatment effectively maintained the coconut water’s color stability, sensory, and physicochemical quality of coconut water. The treatment delayed the enzymatic browning through reducing the PPO and POD activities, preserving the antioxidant capacity through increasing the TPC, TAA, and FRAP. Extending CP treatment time enhanced the sterilization and preservation efficacy, inhibiting shifts in microbial diversity (delaying dominance transitions of *Kosakonia*, *Lactobacillus*, and *Leuconostoc*). Therefore, CP treatment is a potential non-thermal technology able to extend the shelf life of coconut water.

As a non-thermal technology for efficient sterilization, cold plasma has a very good application research effect in fruit and vegetable juices and beverages. It has been proven that it can inhibit the growth of microorganisms and maintain the sensory and physicochemical quality of products. Since there is no change in the flavor of the product after treatment in this paper, the subsequent research needs to study the effect and the mechanism of CP treatment on the taste of the product to achieve the purpose of effectively maintaining the unique flavor quality of the product. The equipment of cold plasma has not been produced a large-scale commercial production, which is an important factor restricting the application of this technology in the food industry. Therefore, it is necessary for researchers, equipment manufacturers, and enterprises to work together to promote equipment development and provide the basis for the commercial application of this technology.

## Figures and Tables

**Table 1:** Changes in cold plasma-treated coconut water ΔE* during storage ^1^.

Storage (d)	Treatment Time (s)			
	0	3	6	12
0	-	0.14 ± 0.06 ^c^	0.18 ± 0.03 ^c^	0.29 ± 0.05 ^c^
3	0.27 ± 0.05 ^c^	0.29 ± 0.04 ^b^	0.21 ± 0.02 ^c^	0.35 ± 0.05 ^b^
6	0.42 ± 0.01 ^b^	0.32 ± 0.03 ^b^	0.32 ± 0.04 ^b^	0.46 ± 0.02 ^b^
9	0.58 ± 0.05 ^b^	0.40 ± 0.05 ^b^	0.42 ± 0.07 ^b^	0.52 ± 0.03 ^b^
12	0.84 ± 0.04 ^a^	0.66 ± 0.04 ^a^	0.79 ± 0.06 ^a^	0.86 ± 0.03 ^a^
15	0.96 ± 0.03 ^a^	0.79 ± 0.06 ^a^	0.84 ±0.06 ^a^	0.94 ± 0.07 ^a^

Note: letters indicate differences between different days.

**Table 2:** Changes in cold plasma-treated coconut water browning index during storage ^2^.

Storage (d)	Treatment Time (s)			
	0	3	6	12
0	0.65 ± 0.00 ^e^	0.76 ± 0.02 ^d^	0.81 ± 0.01 ^d^	0.86 ± 0.03 ^d^
3	0.91 ± 0.01 ^d^	0.90 ± 0.00 ^c^	0.93 ± 0.01 ^c^	0.95 ± 0.02 ^c^
6	0.98 ± 0.00 ^d^	0.94 ± 0.01 ^bc^	0.96 ± 0.02 ^bc^	0.99 ± 0.00 ^c^
9	1.15 ± 0.03 ^c^	0.97 ± 0.01 ^b^	1.00 ± 0.01 ^b^	1.12 ± 0.02 ^b^
12	1.23 ± 0.01 ^b^	1.01 ± 0.03 ^a^	1.09 ± 0.00 ^b^	1.16 ± 0.03 ^b^
15	1.38 ± 0.02 ^a^	1.11 ± 0.03 ^a^	1.20 ± 0.01 ^a^	1.24 ± 0.03 ^a^

Note: letters indicate the differences between different days.

**Table 3:** Alpha diversity index.

Storage (d)	Richness	Chao1	Shannon	Simpson
0	572.67 ± 47.42	572.00 ± 58.04	3.14 ± 0.13	0.07 ± 0.01
3	528.57 ± 31.02	527.67 ± 38.55	3.52 ± 0.19	0.05 ± 0.01
6	385.50 ± 49.21	384.33 ± 60.30	3.45 ± 0.18	0.06 ± 0.03
9	429.90 ± 19.99	428.67 ± 25.32	3.47 ± 0.14	0.06 ± 0.01
12	385.50 ± 37.56	384.00 ± 31.10	3.31 ± 0.22	0.10 ± 0.06
15	462.50 ± 25.37	462.00 ± 26.56	2.19 ± 0.34	0.43 ± 0.05

**Table 4:** The OUTs diversity in cold plasma-treated coconut water samples.

Storage (d)	Phylum	Class	Order	Family	Genus
0	17	24	71	112	185
3	22	36	75	139	249
6	19	23	71	112	195
9	17	28	73	116	207
12	23	38	70	123	202
15	17	30	78	139	384

## Data Availability

Data will be made available on request.
